# Experimental taphonomy of organelles and the fossil record of early eukaryote evolution

**DOI:** 10.1126/sciadv.abe9487

**Published:** 2021-01-27

**Authors:** Emily M. Carlisle, Melina Jobbins, Vanisa Pankhania, John A. Cunningham, Philip C. J. Donoghue

**Affiliations:** School of Earth Sciences, University of Bristol, Life Sciences Building, Tyndall Avenue, Bristol BS8 1TQ, UK.

## Abstract

The timing of origin of eukaryotes and the sequence of eukaryogenesis are poorly constrained because their fossil record is difficult to interpret. Claims of fossilized organelles have been discounted on the unsubstantiated perception that they decay too quickly for fossilization. We experimentally characterized the pattern and time scale of decay of nuclei, chloroplasts, and pyrenoids in red and green algae, demonstrating that they persist for many weeks postmortem as physical substrates available for preservation, a time scale consistent with known mechanisms of fossilization. Chloroplasts exhibit greater decay resistance than nuclei; pyrenoids are unlikely to be preserved, but their presence could be inferred from spaces within fossil chloroplasts. Our results are compatible with differential organelle preservation in seed plants. Claims of fossilized organelles in Proterozoic fossils can no longer be dismissed on grounds of plausibility, prompting reinterpretation of the early eukaryotic fossil record and the prospect of a fossil record of eukaryogenesis.

## Teaser

Taphonomy experiments reveal the potential for a fossil record of the origin of complex life.

## INTRODUCTION

The origin of eukaryotes is among the most formative of events in Earth history, facilitating the emergence of complex multicellular life. However, almost every aspect of eukaryogenesis has proven controversial, not least the sequence of acquisition of eukaryotic characters and the timing of origin of crown eukaryotes (*1*). The last eukaryote common ancestor (LECA) would have had all fundamental eukaryotic characteristics, including a complex cytoskeleton, nucleus, mitochondria, and other membrane-bound organelles, but when and in what order these traits were acquired before LECA is uncertain. The fossil record has been effectively silent on these issues, in part because of the challenge of identifying early fossil eukaryotes, which have conventionally been discriminated on the basis of size, cyst wall complexity, and circumstantial evidence of a cytoskeleton (*2*). Unfortunately, none of these criteria are definitive, since the size distinction from prokaryotes is probabilistic, not deterministic, and an actin cytoskeleton is an ancestral feature of archaea, not a eukaryotic innovation (*3*). Thus, the challenge of discriminating stem from crown eukaryotes, as well as eukaryotes from prokaryotes, appears insurmountable (*4*). Fossilized organelles would provide a more definitive criterion for identifying eukaryote-grade fossils, as well as informing on the evolutionary assembly of eukaryotes and the timing of emergence of the fundamental clades of photosynthetic eukaryotes. While there are many claims of fossilized nuclei and other organelles through the Proterozoic and Phanerozoic [e.g., (*5*–*14*)], their identification is often contentious and many are instead interpreted as collapsed cytoplasmic remains. This null interpretation stems largely from classical experiments in which the cytoplasm of decaying bacteria collapsed into small dense structures that resemble nuclei (*15*, *16*), and it is now commonly held that organelles cannot fossilize because they decay too quickly (*13*). However, there is no experimental evidence to support this view, and there are unequivocal records of intracellular organelles from the recent geologic past (*5*–*10*). Taphonomy experiments characterize patterns of decay, providing an interpretative model for the fossil record. These experiments have previously aided interpretation of Ediacaran Weng’an embryo-like fossils, demonstrating the feasibility of fossilizing embryos and precluding the interpretation of these fossils as giant sulfur bacteria (*17*, *18*). Here, we undertake taphonomy experiments on eukaryotic organelles to complement classical experiments on bacterial-grade cells, to test the general view that organelles decay too rapidly to be fossilized.

## RESULTS

**Fig. 1:**
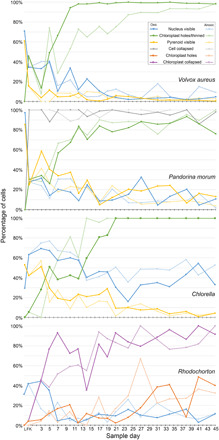
Percentage of algal cells that have characteristics of decay. Acquisition of different features as decay progresses in oxic and anoxic groups. Samples were taken while the algae were alive (L), immediately after death (FK), and every 2 to 3 days for 6 weeks. The development of holes in *Rhodochorton* was particularly noticeable, and so this was measured separate from the collapse of the chloroplast edges. In *P. morum*, the cells themselves collapsed after death.

We performed taphonomy experiments on four species of algae: the unicellular green alga *Chlorella* sp., the colonial green algae *Volvox aureus* and *Pandorina morum*, and the multicellular red alga *Rhodochorton* sp. These species reflect different ecologies and evolutionary grades: freshwater (green algae) versus marine (red alga), unicellularity (*Chlorella*) versus multicellularity, large colony size (*V. aureus*) versus small colony size (*P. morum*), and pyrenoids (green algae) versus none (red alga). Sampling across Rhodophyta and Viridiplantae facilitates a test of consistency among the results. The algae were euthanized in β-mercaptoethanol (BME) to prevent autolysis (*18*) and allowed to decay for 6 weeks in either fresh water (the green algae) or artificial seawater (*Rhodochorton*), reflecting their natural ecology. We performed parallel experiments under oxic and anoxic conditions to control for the effects of aerobic microbial activity; there was no difference in the patterns of decay between the two groups, but anoxic conditions exhibited slower rates in agreement with previous taphonomy experiments (*19*, *20*). Periodic sampling allowed the identification of sequences of decay in the organelles (Fig. 1). The time span of the experiments was chosen to exceed that required for exceptional fossil preservation through phosphate or silica mineral replication, two mineral systems that facilitate fossilization with subcellular fidelity [e.g., (*21*)].

**Fig. 2:**
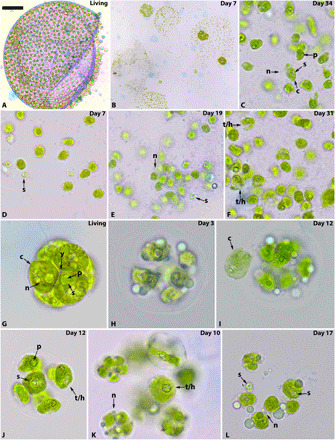
Decay of *V. aureus* and *P. morum*. (**A** to **F**) *V. aureus* and (**G** to **L**) *P. morum*. Living colonies of *V. aureus* (A) show clear cell boundaries and structure, but after death, the colonies display some disaggregation (B). As decay progresses, chloroplasts become less regular in shape (C and E) and develop holes and thin patches (F). Pyrenoids disappear from within the chloroplast, leaving holes ringed by starch grains with no pyrenoid visible (D and E). Some nuclei were still visible weeks after death with no evidence of deformation (C and E). Living colonies of *P. morum* are closely arranged (G), but this collapses after cell death, resulting in the loss of Y-shaped junctions (H). One chloroplast was observed leaving the cell in a cloud (I), but usually, chloroplasts developed holes and thin patches as they decayed (J and K). In late stages of decay, small amounts of green chloroplast were left surrounding the remnants of the starch grain ring (L). Nuclei could still be observed well into decay (K and L). Pyrenoids decayed quickly to leave empty starch grain rings, but this varied within colonies, with some cells still with visible pyrenoids (J and L). n, nucleus; c, chloroplast; s, starch grain ring; p, pyrenoid; y, Y-shaped junction; t/h, thinning/holes within the chloroplast. The number of days postmortem is indicated in the top right corner. Scale bars, 50 μm (A), 91.4 μm (B), 9.1 μm (C), 9.0 μm (D), 9.0 μm (E), 9.1 μm (F), 7.8 μm (G), 6.3 μm (H), 9.1 μm (I), 9.1 μm (J), 9.1 μm (K), and 9.0 μm (L).

Cells of *V. aureus* are broadly the same as those of the closely related *P. morum*, but colonies contain anywhere from 500 to 10,000 cells held in place by a complex network of cytoplasmic bridges and extracellular matrix (Fig. 2A) (*22*). After death, the colonies maintained their general shape for several weeks, although the cells gradually became less evenly spaced and the colonies deformed (Fig. 2B). Some colonies ruptured, while others formed large, misshapen conglomerates (Fig. 2B). Within the cells, the chloroplasts and nuclei remained after 6 weeks, although nuclei usually disappeared before chloroplasts (Figs. 1 and 2, C and E). Pyrenoids were the least decay resistant, although their surrounding starch grain ring persisted (Fig. 2, D and E). Chloroplasts deformed as they decayed, thinning and perforating before fragmenting and disappearing (Fig. 2F), but nuclei were more consistent in appearance (Fig. 2, C and E).

Colonies of *P. morum* are composed of between 4 and 32 cells with Y-shaped junctions (Fig. 2G) (*22*). Each has a single cup-shaped chloroplast occupying most of the volume, one to several pyrenoids, several vacuoles, and a nucleus in the gap left by the shape of the chloroplast (*22*). The colonies started to collapse immediately after death; cells decreased in volume and separated, leading to loss of the Y-shaped junctions (Fig. 2H). Nuclei were still visible and undeformed 6 weeks postmortem, although their abundance decreased (Figs. 1 and 2, K and L). Chloroplasts were the most decay resistant of the organelles analyzed, present in many cells after 6 weeks, but showed evidence of deformation: irregular edges, holes, and thinned areas developed before the chloroplast ultimately fragmented and became indiscernible (Fig. 2, J to L). Pyrenoids were the least decay resistant, decaying within 3 weeks and leaving a hole in the chloroplast. No pyrenoids were observed without the chloroplast, but the starch grain ring that surrounds the pyrenoid was very decay resistant, often observed as an isolated ring after the rest of the cell contents decayed away (Fig. 2, J and L).

**Fig. 3:**
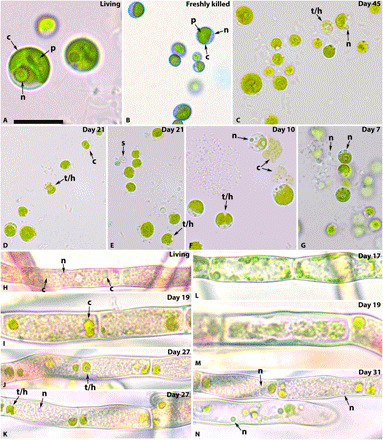
Decay of *Chlorella* sp. and *Rhodochorton* sp. (**A** to **G**) *Chlorella* sp. and (**H** to **N**) *Rhodochorton* sp. Living cells of *Chlorella* (A) show little difference from those immediately after death (B). As decay progresses, chloroplasts collapse, becoming less regular in shape (C and D). Pyrenoids disappear quickly, leaving empty starch grain rings (E), while chloroplasts thin and develop holes (D to F). Nuclei could still be observed in some cells (C, F, and G). In some cases, the chloroplast can escape the cell (F). Living *Rhodochorton* cells (H) also show little difference from those immediately after death, although chloroplasts collapse and deform quickly (I). Holes develop within the chloroplasts (J and K) and in later stages of decay can occasionally conglomerate along the cell walls along with the cytoplasmic contents (L) or pull away from the cell wall (M). Nuclei can still be observed in some cells, even when much of the cytoplasm is gone (K and N). The number of days postmortem is indicated in the top right corner. Scale bars, 10 μm (A), 18.7 μm (B), 15 μm (C), 18.5 μm (D), 18.4 μm (E), 15.8 μm (F), 18.7 μm (G), 12.6 μm (H), 11.8 μm (I), 14.9 μm (J), 15.4 μm (K), 14.1 μm (L), 10.4 μm (M), and 14.8 μm (N).

Unicellular *Chlorella* is similar to component cells of colonial *Pandorina* and *Volvox*: A large cup-shaped chloroplast with a single pyrenoid occupies most of the cell, with a nucleus and vacuoles in the gap (Fig. 3A) (*22*). Immediately after death, cells showed little or no evidence of change (Fig. 3B). Nuclei were visible throughout the 6 weeks and showed no evidence of degradation (Fig. 3, C, F and G). Pyrenoids were the least decay resistant, while chloroplasts were the most decay resistant (Fig. 1). Chloroplasts occasionally escaped the cell during decay if the cell ruptured early (Fig. 3F). Otherwise, chloroplasts collapsed, producing irregular edges before developing holes, thinning, fragmenting, and disaggregating (Fig. 3, C to F).

The filamentous red alga *Rhodochorton* has cells with a central nucleus and two to three discoid chloroplasts that do not have pyrenoids (Fig. 3H) (*23*). The color and texture of the cytoplasm often obscure view of the nucleus, but nuclei could still be observed in some cells 6 weeks postmortem (Fig. 3, K and N). However, chloroplasts collapsed quickly, as evidenced by the irregular edges and discoid shapes, before forming holes and fragmenting (Fig. 3, I to K). These deformed chloroplasts remained in the cells for the full 6 weeks. The holes that developed were often in the center of the chloroplast, resulting in ring-shaped remains, but could also form along the edges (Fig. 3, J and K). As decay progressed, the cytoplasmic contents sometimes condensed along the edges of the cell, with fragments of chloroplast within (Fig. 3, L and M).

## DISCUSSION

Claims of ancient fossilized intracellular organelles have generally been rejected, despite similarity in size, shape, and locus to organelles in living eukaryotes, on the basis of the following: (i) The composition of nuclei and other organelles (water, proteins, and nucleic acids) indicates that they will degrade rapidly after death, and (ii) if one organelle is preserved, then others should also (*13*). Our taphonomy experiments were designed not to simulate fossilization but, rather, to determine whether eukaryotic organelles persist postmortem as physical structures on a time scale compatible with permineralization, mineral replication, or stabilization as organic remains. Fossilization can progress rapidly: Phosphatization and silicification can occur within weeks of death (*21*).

Our experimental results demonstrate that organelles undergo broadly the same patterns of decay over similar time scales in all four algal species. Nuclei were resistant to notable deformation during decay, whereas chloroplasts underwent extensive changes: Irregular edges, holes, thin patches, and fragmentation occurred before full disintegration. However, chloroplasts, in particular, and nuclei, to a lesser extent, could still be observed in cells 6 weeks after death. The decrease in frequency of nuclei over decay time may be due to chloroplast collapse in the green algae, obscuring or engulfing nuclei, and the rough texture of the cytoplasm in *Rhodochorton*, which obscures identification of nuclei even in living cells. Pyrenoids disappeared quickly, but evidence of their presence remained in the form of the hole within the starch grain ring, often the last remaining structure in a decaying cell. These results agree with previous studies using *Allium cepa* (onion), which showed that nuclei can persist as physical structures on time scales compatible with their mineral replication (*21*, *24*), although we observed no deformation of nuclei. Given that our experimental models include marine and freshwater species, unicellular and multicellular species, as well as representatives of two of the fundamental scions of Archaeplastida (Rhodophyta and Viridiplantae), it is reasonable to conclude that these observations can be generalized. The available experimental evidence indicates that claims of fossil eukaryotic organelles should be taken seriously.

### Comparison to uncontentious fossil organelles

**Fig. 4:**
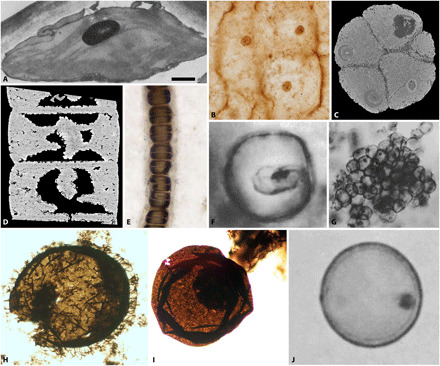
Fossil organelles. (**A**) Fossil of a *Zelkova* leaf from the Miocene Succor Creek Formation showing a chloroplast adpressed to the cell wall, with grana stacks visible and a densely stained starch grain. Transmission electron micrograph after a carbohydrate cytochemical analysis; image from (*29*) courtesy of Karl Niklas, Cornell University. (**B**) Segment of the stem of a royal fern from Jurassic deposits with clear nuclei in each cell. Light micrograph; image courtesy of Benjamin Bomfleur, University of Münster. (**C**) Tomographic virtual section through *Megasphaera*, a fossil from the Weng’an Biota that is suggested to have nuclei. Data courtesy of (*35*). (**D**) Intracellular structures in the holotype of *R. chitrakootensis* have previously been interpreted as pyrenoids but are unlikely to be so. Tomographic reconstruction; data from (*41*) courtesy of Stefan Bengtson, Swedish Museum of Natural History. (**E**) *Bangiomorpha pubescens*, currently considered the oldest crown eukaryote. Light micrograph; image courtesy of Nicholas Butterfield, University of Cambridge. (**F**) *Caryosphaeroides* and (**G**) *Glenobotrydion* from the Bitter Springs Biota have been suggested to be early eukaryotes with putative nuclei or chloroplasts. Light micrographs; images from (*38*) courtesy of SEPM. (**H**) *Shuiyousphaeridium* and (**I**) *Dictyosphaera* from the Ruyang Group, putative early eukaryotes with intracellular structures suggested to be nuclei or collapsed cellular contents. Light micrographs; images courtesy of Shuhai Xiao, Virginia Tech. (**J**) *Leptoteichos* from the Gunflint Iron Formation with a putative nucleus. Light micrograph; image from (*45*) courtesy of SEPM (Society for Sedimentary Geology) (SEPM). Scale bars, 1.4 μm (A), 25.6 μm (B), 250 μm (C), 38.2 μm (D), 25 μm (E), 3.3 μm (F), 13.8 μm (G), 14.3 μm (H), 25.3 μm (I), and 3.5 μm (J).

There are numerous compelling claims of fossil nuclei, chloroplasts, and even mitochondria in Cenozoic plant remains that preserve histological detail of organelle structure (*6*–*10*, *25*), and some of which even remain biochemically reactive to specific histological stains (*6*, *9*, *10*). In mummified Eocene *Metasequoia* leaves, the least degraded chloroplasts had clear thylakoid membranes and grana stacks, while the more degraded specimens showed evidence of chloroplast fragmentation, as seen in our experiments (*9*). Similarly, amber has yielded exquisitely preserved chloroplasts that are almost indistinguishable from living chloroplasts, with no loss of shape or structure (*6*, *25*). Miocene angiosperm leaves (Fig. 4A) from the *Clarkia* beds of Idaho (USA) are among the best studied, revealing that chloroplasts and cell walls are more decay resistant than nuclei since they are present in the fossils more frequently (*26*, *27*). However, nuclei have been described from the stem of a Jurassic fern (Fig. 4B) (*5*) and within Carboniferous gymnosperm pollen (*28*), demonstrating that they can be preserved. These empirical observations are consistent with our experimental results: Chloroplasts are the most decay resistant of organelles, but nuclei persist for a considerable length of time postmortem. Mode of preservation has also played a role in preserving these organelles; the *Clarkia* beds were preserved by volcanic ash, leading to desiccation before preservation as organic fossils (*29*), while the Jurassic fern was permineralized rapidly by hydrothermal vents (*5*). The less well-preserved Carboniferous gymnosperm nuclei were from coal balls that would not have fossilized as rapidly as the fern or the Miocene gymnosperms and angiosperms (*28*).

### Comparison to contentious claims of fossil organelles

#### Weng’an embryoids: Bacteria, holozoans, or algae?

Intracellular structures in Weng’an phosphatic embryo-like fossils (Fig. 4C) have been central to debate over their affinity. The interpretation of intracellular structures as nuclei precludes a bacterial interpretation (*30*), favoring affinity with metazoans (*31*) or nonmetazoan holozoans (*32*). Claims that the intracellular structures are too large to be nuclei and that they are preserved in late mineralization phases, after the decay of original biological structures (*33*, *34*), have already been refuted (*12*, *35*). However, the nucleus interpretation has been rejected principally on the basis of plausibility and the expectation that other organelles should also be preserved (*36*). The algal taphonomy experiments demonstrate that nuclei persist postmortem on a time scale compatible with phosphatization (*21*), and the absence of chloroplasts is further evidence against an algal interpretation.

#### Bitter Springs: Organelles or shrunken cytoplasm?

The assumption that nuclei (and by extension other organelles) cannot be preserved in fossils rests in bacterium taphonomy experiments conducted to test claims of eukaryotes in the ~830–million year (Ma) Bitter Springs Formation, Australia (*15*, *37*). Intracellular structures in *Caryosphaeroides*, composed of a pale outer ring surrounding a darker inner spheroid (Fig. 4F), were interpreted as a nucleus (*38*); diffuse structures around a dark, angular central structure in *Glenobotrydion* were interpreted as chloroplasts surrounding a pyrenoid (*39*) or nuclear residues (Fig. 4G) (*40*). Taphonomy experiments demonstrated that bacterial cells can produce cytoplasmic collapse structures that resemble nuclei, leading to the null interpretation of the Bitter Springs intracellular structures as taphonomic artifacts of bacterial cells (*15*). However, our complementary experiments using algae show that the organelle interpretations cannot be rejected so easily; the structures in *Caryosphaeroides* and *Glenobotrydion* could be nuclei or chloroplasts but are unlikely to be pyrenoids on the basis of the relative decay resistance of these organelles. The intracellular structures in *Glenobotrydion* resemble decayed remains of a chloroplast surrounding the remnants of a pyrenoid, while more data are needed on the consistency of size, number, shape, and locus of the intracellular structures in *Caryosphaeroides* to aid their interpretation.

#### Rafatazmia: The earliest red alga?

*Rafatazmia chitrakootensis*, from the ~1600-Ma Tirohan Dolomite of India, has been interpreted as a rhodophyte and therefore the oldest crown eukaryote (*41*). Suspended within comparatively large eukaryote-scale cells, there is sometimes a single large, central, rhomboidal structure or several smaller structures located near the septa between cells, interpreted as pyrenoids and pit plugs, respectively (Fig. 4D) (*41*). Our experiments indicate that pyrenoids would not be present ordinarily without the surrounding chloroplast. Preservation probability does not always correlate with decay resistance (*42*). However, the structures in the holotype wholly fill the cell and are not readily rationalizable with a chloroplast, nucleus, or their degraded remains. Furthermore, many of these purported intracellular structures are irregular in shape and size and appear to be mineralized in the same phase as void-filling cement, with the exception of the holotype. There is not sufficient evidence to identify pit plugs within the cells; without pit plugs, there is little support for a rhodophyte affinity, and the oldest definitive crown-group eukaryote is therefore *Bangiomorpha*, a silicified filamentous fossil from the ~1047-Ma Hunting Formation of Canada (Fig. 4E) (*43*, *44*). Despite being well preserved, placement of *Bangiomorpha* within Bangiaceae is not due to any subcellular detail but its general simplicity and the arrangement of wedge-shaped cells in multiseriate filaments (*43*); this is insufficient to justify a Bangiaceae or crown-rhodophyte affinity. There are no organelles obviously preserved in the fossils, reflecting considerable decay leaving only recalcitrant cell walls.

#### The oldest eukaryotes: Nucleated or not?

Unicellular *Dictyosphaera* and *Shuiyousphaeridium* (~1700-Ma Ruyang Group of China) are the oldest widely accepted eukaryotic fossils, often preserved associated with intracellular structures that have been compared to nuclei (Fig. 4, H and I) (*13*). However, despite their consistency of size, locus, number, and shape, the nucleus interpretation has been rejected on the basis of plausibility and the absence of other organelles; they are instead interpreted as contracted cytoplasmic contents (*13*). Our experimental results demonstrate that the nucleus interpretation cannot be rejected, as it has been, on taphonomic grounds and that the absence of chloroplasts may simply reflect the fact that *Dictyosphaera* and *Shuiyousphaeridium* were not photosynthetic eukaryotes. The miniscule size, low abundance, and need for staining in living cells for observation suggest that mitochondria are unlikely to be identifiable in fossil material that is not amenable to transmission electron microscopy analysis or staining. *Leptoteichos golubicii* [1880-Ma Gunflint Iron Formation, Canada (*45*)] is an even older candidate eukaryote with intracellular structures originally interpreted as collapsed cytoplasmic contents (Fig. 4J); the authors were reluctant to accept a nucleus and eukaryote interpretation because of its great geologic age. These structures strongly resemble the putative nuclei in *Caryosphaeroides* but, similarly, they require detailed characterization of size, locus, number, and shape consistency before a definitive conclusion can be reached.

### Implications for elucidating early eukaryote evolution

Growing evidence supports the two-domain tree of life, with Eukarya unusually having two stem lineages that arise from their α-proteobacterial and archaeal relatives (*46*). Although there can be little hope of palaeontological insight into the former before its coalescence with the archaeal lineage, our experiments demonstrate that there is promise that aspects of the process of eukaryogenesis relating to the origin of organelles may be preserved in the fossil record. However, while it may be possible to distinguish eukaryote-grade fossils, for instance with a preserved nucleus, it will remain challenging to rationalize whether these represent stem or crown eukaryotes. Hence, evidence of preserved nuclei in *Dictyosphaera* and *Shuiyousphaeridium* provides definitive evidence of their eukaryote-grade organization, supplementing circumstantial evidence of an actin skeleton. They provide the oldest definitive evidence of total-group eukaryotes, but definitive evidence of the divergence of crown eukaryotes must rest with the preservation of secondary organelles, specifically chloroplasts and pyrenoids, that is before evidence for the emergence of fungi and metazoans. At present, this record rests with ~1047-Ma *Bangiomorpha* (*44*). However, our experiments provide a basis for pushing back the record of both total group and crown eukaryotes based on the preservation of nuclei, chloroplasts, and pyrenoids, illuminating the time scale and pattern of eukaryogenesis and diversification.

## MATERIALS AND METHODS

The algae were obtained from Sciento.co.uk and immediately sampled to confirm species identification and provide baseline images of the living algae. The algae were then euthanized in 300 mM BME for a minimum of 24 hours, providing time for the cells to settle to the bottom of the tubes. Excess BME was removed with a pipette, and the algae were rinsed in tap water for the freshwater species and artificial seawater for *Rhodochorton*. Samples were taken immediately after euthanization. Each species was then divided into 32 1-ml tubes and separated into two experimental groups: the “oxic” group, with the lid of the tube removed and water topped up to prevent desiccation, and the “anoxic” group, where the lid remained sealed until sampling. The addition of water to the oxic group may have affected the chemical gradients surrounding the algae, and mechanical damage may have occurred while the water was being added. However, since there was little difference between the rates of decay between the oxic and anoxic groups and no difference in the patterns of decay, these appear to have had negligible effects on the decay of the algae. The tubes were then placed in a dark Scientific Laboratory Supplies Ltd. (SLS) incubator at room temperature (17.5°C) to control for effects of different temperatures on the speed of decay and sampled every 2 to 4 days for 6 weeks.

To take samples, a small volume of the algae that had settled to the bottom of the tube was removed using a 1-ml Pasteur pipette and transferred to a microscope slide. Tubes were only sampled once, providing 16 samples across the 6 weeks of the experiment. The tip of a pipette was then dipped in 1% methylene blue and mixed into the algae to enhance the contrast, although this was not necessary for *Rhodochorton* because of its natural coloring. The samples were examined under an Axioskop 40 light microscope with a Leica DFC295 camera. Images were taken of colonies and individual cells using ×10, ×20, ×40, and ×100 lenses and the Leica Application Suite version 4.2.0. Conventional adjustments of color levels, contrast, and brightness were performed on the images using Adobe Photoshop CC 2014 version 2.2.

For each sample, a proportion of random cells (around 100 to 160 cells for *Chlorella* and *V. aureus* and around 40 cells for *Rhodochorton* and *P. morum*) were scored on the basis of whether the nucleus was visible; whether the chloroplast had holes and/or had collapsed; and, in the species with pyrenoids, whether the pyrenoid was visible within the starch grain ring. The numbers of cells counted were based on the abundance of the cells in the images taken during sampling; because of the large size of the colonies of *V. aureus* and the small size and abundance of cells of *Chlorella*, more cells were visible in each photograph. *Rhodochorton* cells are much larger and were therefore less abundant in the photographs, and *P. morum* colonies are both limited in cell number and overlapping, resulting in fewer visible cells. The data on decay characteristics were analyzed using Microsoft Excel.

## SUPPLEMENTARY MATERIALS

Supplementary material for this article is available at http://advances.sciencemag.org/cgi/content/full/7/5/eabe9487/DC1
